# Total knee arthroplasty: does ultra-early physical therapy improve functional outcomes and reduce length of stay? A retrospective cohort study

**DOI:** 10.1186/s13018-024-04776-y

**Published:** 2024-05-10

**Authors:** Lynn Thwin, Brian Rui Kye Chee, Yan Mei Yap, Kelvin Guoping Tan

**Affiliations:** 1 Department of Orthopaedic Surgery, Tan Tock Seng Hospital, Singapore, Singapore; 2 Department of Physiotherapy, Tan Tock Seng Hospital, Singapore, Singapore

## Abstract

**Background:**

The Enhanced Recovery After Surgery (ERAS) Society recommends that after total knee arthroplasty (TKA), patients should be mobilized early. However, there is no consensus on how early physical therapy should be commenced. We aim to investigate whether ultra-early physical therapy (< 12 h postoperatively) leads to better outcomes.

**Methods:**

This is a retrospective cohort study of 569 patients who underwent primary TKA from August 2017 to December 2019 at our institution. We compared patients who had undergone physical therapy either within 24 h or 24–48 h after TKA. Further subgroup analysis was performed on the < 24 h group, comparing those who had undergone PT within 12 h and within 12–24 h. The outcomes analyzed include the Oxford Knee Scoring System score, Knee Society Scores, range of motion (ROM), length of stay (LOS) and ambulatory distance on discharge. A student’s t test, chi-squared test or Fisher’s exact test was used where appropriate, to determine statistical significance of our findings.

**Results:**

LOS in the < 24 h group was shorter compared to the 24–48 h group (4.87 vs. 5.34 days, *p* = 0.002). Subgroup analysis showed that LOS was shorter in the ultra-early PT (< 12 h) group compared to the early PT (12–24 h) group (4.75 vs. 4.96 days, *p* = 0.009). At 3 months postoperatively, there was no significant difference in ROM, ambulatory distance or functional scores between the < 24 h group and 24–48 h group, or on subgroup analysis of the < 24 h group.

**Conclusion:**

Patients who underwent physical therapy within 24 h had a shorter length of stay compared to the 24–48 h group. On subgroup analysis, ultra-early (< 12 h) physical therapy correlated with a shorter length of stay compared to the 12–24 h group (4.75 vs. 4.96 days, *p* = 0.009) - however, the difference is small and unlikely to be clinically significant. Ultra-early (< 12 h) physical therapy does not confer additional benefit in terms of functional scores, ROM or ambulatory distance. These findings reinforce the importance of early physical therapy after TKA in facilitating earlier patient discharge.

## Introduction

Total knee arthroplasty (TKA) is the mainstay of treatment for late stage osteoarthritis, and is being performed with increasing frequency each year.

Multiple preventive strategies have been documented in the literature - surgical and nonsurgical - in an attempt to slow the progression of knee osteoarthritis [1–3]. However, with many countries facing an aging and more active population, the incidence of severe knee osteoarthritis requiring joint replacement continues to increase. [4, 5]

The Enhanced Recovery After Surgery (ERAS) Society recommends that TKA patients should be mobilized as early as possible after surgery to aid quicker recovery [6]. Studies have suggested that early physical therapy helps to improve functional outcomes, range of motion (ROM) as well as in-hospital length of stay (LOS) [7–12]. In addition to improving functional outcomes, safely reducing the hospital LOS also helps with reducing healthcare costs as one of the main cost contributors in a TKA is the length of inpatient stay [13, 14]. 

Early initiation of physical therapy (PT) post-op has been proposed as one of the ways to reduce LOS [7, 15]. It has been shown that PT can be safely initiated as early as post operative day zero (POD 0) [15]. Some studies showed that early initiation of PT may allow a more efficient and productive PT session on subsequent days resulting in better ROM and functional outcomes [9, 11]. Early initiation of PT can also reduce the development of complications such as deep vein thrombosis, pulmonary emboli, chest infections and urinary retention [16]. However, the definition of early PT varies from study to study. Some studies use the number of post-operative days in their definition, while others use the number of hours from the time of surgery.

In addition, while many of the studies have concentrated on the LOS as well as safety aspects of early rehabilitation, only one study has looked at the functional outcomes associated with early PT [11]. The benefit for patients in terms of functional outcomes has not been adequately studied in the existing literature.

This study aims to investigate how the timing of physical therapy affects the functional outcomes as well as LOS of patients undergoing TKA.

## Methods

This is a retrospective cohort study of 569 patients who underwent primary TKA between August 2017 and December 2019 at our institution. Institutional review board approval was obtained before performing this study (NHG DSRB 2020/00095). The data was prospectively collected from our institution’s existing knee registry.

The inclusion criteria comprises:

1. Patients who underwent unilateral TKA.
2. Patients who were discharged home.
3. Patients who commenced physical therapy within 48 h of their surgeries.

Patients who underwent unicompartmental knee arthroplasty, revision knee arthroplasty (for any cause), or bilateral total knee arthroplasty were excluded. Patients discharged to community rehabilitation centers were also excluded.

Patient demographics and surgical data were collected for all patients, including age, gender, body mass index (BMI), side (left or right), comorbidities and duration of operation.

All the patients were admitted to the hospital on the day of surgery. The length of stay was calculated from their date of admission to the date of discharge in terms of days. The timing of each case was determined by the availability of operative resources and the primary surgeon’s preference.

The TKAs were performed using medial parapatellar approaches. All the patients received either a peripheral nerve block or periarticular injection intra-operatively. The type of anesthesia was also recorded as spinal anesthesia or general anesthesia. After the surgery, all patients were first transferred to the post-anaesthesia care unit (PACU) and thereafter to the general wards for inpatient care.

Patients were selected for physical therapy based on availability of the physiotherapists and timing of the surgery. Patients who arrived in the ward from PACU after office hours commenced their physical therapy on the following day after their surgery. All patients received physical therapy within 48 h from the time of their respective surgeries.

The aims of physical therapy were the same regardless of whether it was commenced on the day of surgery or the subsequent days. The physical therapists determined the length and frequency of therapy sessions according to the needs of individual patients.

The patients were started on continuous passive motion (CPM) on the day of their surgery. The physical therapists would assist the patients to sit over the edge of bed, and also initiate active and active assisted knee range of motion (ROM) exercises. As the patients progressed, they would be taught transfers, started on gait training, stair climbing and activities of daily living exercises.

The patients were deemed safe for discharge after they were able to ambulate with a walking aid, clear ground level obstacles safely, have adequate pain control and a clean and dry surgical site.

The amount of distance covered on the day of discharge as well as their knee ROM were recorded.

Functional outcomes were measured using the Oxford Knee Score (OKS) and Knee Society Score (KSS) and KSS (function), and were collected until three months after the operation.

The time taken from the end of surgery to the start of physical therapy was recorded. During the analysis, the patients were grouped into those that commenced physical therapy within 24 h and 24–48 h after operation. The < 24 h group was also further subdivided to < 12 h and 12–24 h. We defined patients who received PT within 24 h as early PT and those who received within 12 h as ultra-early PT. Differences in outcomes were compared between < 24 h and 24–48 h groups, and a subgroup analysis of the < 24 h group was also performed to determine whether ultra-early physical therapy has an effect on the outcomes measured.

### Statistical methods

Descriptive statistics of the demographic data and outcome variables were calculated. Numerical variables were presented as mean ± standard deviation or median (IQR) and Student’s t-test was used when appropriate. Categorical variables were presented as numbers and percentage and Chi-square test or Fisher Exact test was used when appropriate. A two tailed significance level of 0.05 was used for all the tests. All statistical analyses were conducted using IBM SPSS Statistics 19.

## Results

A total of 1312 patients underwent TKA during the specified period. Of these, 569 patients had unilateral surgery and were discharged home, and were hence included in the study. 478 patients underwent PT within 24 h and 91 patients within 48 h. There were no significant differences in age, gender, BMI and preoperative hemoglobin levels, pre op range of motion as well as functional scores between the two groups (Table 1).

**Table 1:** Baseline characteristics

	< 24 h (*n*=478)	24–48 h (*n*=91)	*P*-value
	Mean ± SD	Mean ± SD	
Age (Years)	67.59± 7.81	68.43±7.68	0.498
Gender (Male: Female ratio)	173:305	35:56	0.722
BMI (kg/m^2^)	27.32 ± 4.75	27.74± 5.01	0.615
Anaesthesia (Spinal: GA ratio)	334:144	65:26	0.804
Preoperative hemoglobin	13.39 ± 1.31	13.23± 1.41	0.284
Operative Time (minutes)	94.71± 31.72	90.51± 27.90	0.339
Pre op PROM	102.67± 18.29	105.38±16.72	0.174
Pre op Oxford	26.31± 6.90	27.58± 12.55	0.652
Pre op KSS Function	50.89± 17.77	49.61±18.10	0.436
Pre op KSS	44.16± 15.11	45.13± 15.12	0.543

There was no statistical significance between the groups with regards to co-morbidities (Table 2).

**Table 2:** Co-morbidities. IHD – ischaemic heart disease, COPD – chronic obstructive pulmonary disease, CVA – cerebrovascular accident, TIA – transient ischaemic attack

	< 24 h (*n*=478)	24–48 h (*n*=91)	*P*-value
	Mean ± SD	Mean ± SD	
Diabetes	95 (19.9%)	15 (16.5%)	0.877
Hypertension	267 (55.9%)	48 (52.7%)	0.669
Hyperlipidaemia	219 (45.8%)	39 (42.9%)	0.791
Asthma	25 (5.2%)	3 (3.3%)	0.783
IHD	25 (5.2%)	5 (5.5%)	0.788
COPD	2 (0.4%)	0 (0%)	1.00
Prev Cancer	23 (4.8%)	4 (4.4%)	1.00
Inflammatory Arthritis	12 (2.5%)	4 (4.4%)	0.270
Smoker	9 (1.9%)	0 (0%)	0.369
CVA / TIA	23 (4.8%)	6 (6.6%)	0.411
Osteoporosis	15 (3.1%)	3 (3.3%)	0.736

There was significant reduction in LOS in the < 24 h group compared to 24–48 h group (4.87 vs. 5.34 days, *p* = 0.002). There were no significant differences in other functional outcome measures between the 2 groups. (Tables 3 and 4). There was no increase in the complication rates between the two groups of patients.

**Table 3:** Length of stay (LOS), Ambulatory distance, Passive range of motion (PROM) and Functional outcome scores on discharge and 3 month post operative

Outcomes	< 24 h (*n*=478)	24–48 h (*n*=91)	*P*-value
	Mean ± SD	Mean ± SD	
Length of stay (days)	4.87±2.10	5.34± 1.84	0.002
Ambulatory distance (meters)	31.02± 24.15	28.44± 16.76	0.626
PROM on discharge	90.27± 14.78	87.85± 15.55	0.236
PROM @ 3mth	107.12± 15.39	110.27± 14.50	0.087
Oxford Knee score 3 month	40.19±5.45	40.88 ± 4.27	0.464
KSS Function 3 month	72.57± 16.88	73.13± 16.67	0.949
KSS 3 month	84.31± 12.33	85.7± 12.03	0.285

**Table 4:** Interval change in Function outcome scores. KSS = Knee Society Score

Outcomes	< 24 h (*n*=478)	24–48 h (*n*=91)	*P*-value
	Mean ± SD	Mean ± SD	
Oxford Knee Score 3 month - Preop	13.3± 10.27	11.95± 14.62	0.609
KSS Function 3 month - Preop	21.58± 23.91	23.54± 23.01	0.573
KSS 3 month - Preop	39.52± 20.18	39.63±21.28	0.994

A subgroup analysis was performed for the < 24 h group to determine whether patients who received ultra-early physical therapy (< 12 h) have shorter LOS as well as other functional outcomes compared to those who underwent early physical therapy (12–24 h). The ultra-early physical therapy group showed a shorter length of stay (4.75 vs. 4.96 days, *p* = 0.009). There was no significant difference in PROM, ambulatory distance at discharge and functional outcome between the ultra-early PT and early PT group (Table 5).

**Table 5:** Subgroup analysis of patients who underwent physical therapy within 24 h and their outcome scores

Outcomes	< 12 h (*n*=216)	12–24 h (*n*=262)	*P*-value
	Mean ± SD	Mean ± SD	
Length of stay (days)	4.75± 2.32	4.96± 1.89	0.009
Ambulatory distance (meters)	30.22±19.83	31.68 ± 27.23	0.923
PROM on discharge	90.72± 14.72	89.89± 14.85	0.513
PROM @ 3mth	108.22± 14.49	106.19± 16.08	0.160
Oxford Knee score 3 month	40.40±5.65	40.00± 5.29	0.215
KSS Function 3 month	73.59± 16.38	71.68±17.30	0.233
KSS 3 month	85.48± 11.36	83.31±13.03	0.088
Oxford Knee Score 3 month - Preop	13.93± 10.28	12.73± 10.26	0.13
KSS Function 3 month - Preop	22.65± 24.03	20.64±23.83	0.587
KSS 3 month - Preop	41.58±18.89	37.79± 21.09	0.079

## Discussion

Early mobilization is an integral part of enhanced recovery after surgery (ERAS) [17]. The ERAS society recommends that patients should be mobilized as soon as possible after surgery - this helps to reduce the length of stay and counteract the adverse physiological effect of prolonged bed rest [6]. In current literature, studies have shown that early mobilization or rehabilitation after total knee arthroplasty improves functional outcomes as well as reduces the length of hospital stay [7, 11, 12, 18, 19].

Our study’s findings correlate with that of previous studies which have found that early mobilization on POD 0 following TKA leads to shorter LOS [13, 15, 20]. Chen et al. found in their prospective cohort study that patients who ambulated with PT on POD 0 had a shorter LOS than those patients who did not [15]. Similarly, Den Hertog et al’s randomized controlled trial showed that patients who were in a POD 0 fast-track rehabilitation program had a shorter LOS compared to standard rehabilitation programs in their randomized control trial [7]. In our study, all patients received PT within POD1. Patients in the ultra-early group received PT on POD0 while patients in the early (12–24 h) group and > 24 h group had PT on POD 1. Although both ultra-early and early physical therapy showed benefits in terms of LOS, our results show that there is diminishing benefits to ultra-early physical therapy (LOS: 4.75 vs. 4.96 days comparing < 12 h and 12–24 h) compared to that of early physical therapy (LOS: 4.87 vs. 5.34 days comparing < 24 h and 24–48 h). Although the ultra-early physical therapy (< 12 h) group showed a lower LOS compared to the 12–24 h group, the small difference (4.75 vs. 4.96 days) is unlikely to be clinically significant. In the healthcare setting where resources and manpower are finite and limited, it would be necessary to weigh the benefits of a slightly reduced LOS from ultra-early physical therapy against manpower and resource limitations.

In addition, the recorded mean LOS in our study is longer than what has been reported in previous literature (Bohl et al.: median of 32 h for POD0 PT and 31 h for POD1 PT; Chen et al.: mean of 2.8 days for POD0 PT and 3.7 days for POD1 PT). There are 2 main factors that could have contributed to our longer LOS, which are: the higher average age of our patients, and the difference in criteria for safe discharge by our physical therapists. The average age of our patients’ population (67.7 years) was several years older than patients in other studies (Bohl et al. 63.7 years, Chen et al. 62.3 years) [8, 15]. This could contribute to slower recovery from the initial surgery as well as a slower rehabilitation process. Secondly, there is a strict criteria for safe discharge from the physical therapists at our institution. These include the ability to ambulate with a walking aid, clear ground level obstacles safely, achieve adequate pain control and a clean and dry surgical site. In the study by Bohl et al. 2019, there was no explicit criteria that was mentioned for safe discharge. An arbitrary term of being “cleared” by the physical therapist was used to denote when a patient was safe to be discharged from the acute hospital ward [8]. Chen et al’s study defined their discharge criteria, which included being able to ambulate 100ft and being able to climb up and down stairs. However, the amount of assistance required and the type of mobility aid required to achieve these discharge criteria were not mentioned. In addition, patients who were discharged to skilled nursing facilities and rehabilitation facilities were also included in their study. We have observed that in our local population, a significant percentage of patients have the expectation of only being discharged when they are close to independent ambulation. This would have a great impact on the LOS as a larger number of PT sessions would be needed to get these patients back to or close to their premorbid function.

Our data finds that at 3 months follow-up, there was no significant difference in change in OKS and KSS functional scores - differing from results from other studies in the current literature. Larsen et al. conducted a randomized controlled trial to investigate the effects of accelerated rehabilitation and physical therapy after arthroplasty. Their study involved a heterogenous group of 87 patients who had undergone either total hip arthroplasty (THA), total knee arthroplasty (TKA) or unicompartmental knee arthroplasty (UKA). They found that at 3 months follow-up, there was a significant gain in quality of life (QOL) using the EQ−5D score compared to baseline [11]. However, a subgroup analysis of TKA patients did not reveal any significant difference in the functional outcomes - it appears that their findings were skewed by significantly better results experienced by the THA patients who had undergone early physical therapy. Furthermore, the intervention group not only received earlier physical therapy, but they were also provided with pre-operative education and longer physical therapy sessions. Patients in the intervention group were also cohorted in the same ward. Thus, while Larsen et al’s study shows better functional outcomes after earlier intervention, it is difficult to isolate these effects to merely early initiation of physical therapy.

While other studies have found an increase in ROM with early initiation of physical therapy, our study did not find similar results [9, 10]. Labraca et al’s randomized controlled trial had patients placed into two groups - those receiving physical therapy either before 24 h or after 24 h postoperatively. Patients who received physical therapy within 24 h showed significantly better LOS, ROM and gait scores. While our study found a similar correlation between earlier physical therapy and a shorter LOS, it did not find that earlier physical therapy led to increased ROM at discharge.

There are several strengths to our study. Firstly, the data was collected prospectively, which reduces the risk of recall bias. Secondly, other studies investigating the early initiation (POD 0 vs. POD 1) of PT mainly use LOS as an outcome factor. The present study not only uses LOS, but also compares the ROM, ambulation distance as well as early functional outcome score (3 months) to determine the difference in outcomes when PT is initiated ultra-early, early or after 24 h from surgery. Thirdly, our sample size (*n* = 569, ultra-early PT *n* = 216, early PT *n* = 262, > 24 h *n* = 91) is relatively large when compared to other recent studies. Finally, our study isolates time to PT as the independent variable between treatment groups - other than this, there were no differences in intervention across treatment groups.

We recognise several limitations to our study. Firstly, the patients were not randomized and patients who were not well medically, not motivated to undergo PT or underwent surgeries later in the day were self-selected to be in the 12–24 h group or 24–48 h group. This might result in selection bias in the study. Secondly, our study focuses on patients who were discharged home. Over half of our TKA patients are discharged to subacute facilities for further rehabilitation prior to being discharged home - our study does not address the effects of earlier physical therapy on this significant patient population. Thirdly, LOS was defined as the time between admission and discharge at our institution. Besides the effects of earlier physical therapy, other non-related issues such as medical complications, patient preference and administrative discharge paperwork may also influence a patient’s length of stay and obscure the differences due to earlier physical therapy.

## Conclusion

Our study shows that early commencement of physical therapy within 24 h is important in reducing the LOS (4.87 vs. 5.34 days comparing < 24 h and 24–48 h).

Ultra-early physical therapy (< 12 h) confers additional benefit in terms of length of stay (4.75 vs. 4.96 days, *p* = 0.009) compared to the 12–24 h group - however, the difference is small and unlikely to be clinically significant. Ultra-early (< 12 h) physical therapy does not confer additional benefit in terms of functional scores, ROM or ambulatory distance.

These findings reinforce the importance of early physical therapy after TKA in facilitating earlier patient discharge.

Where resources permit, physical therapy should be initiated on the day of operation, and ideally as soon as the patient returns to the ward.

## Data Availability

Data is provided within the manuscript or supplementary information files.
